# The CEPH aging cohort and biobank: a valuable collection of biological samples from exceptionally long-lived French individuals and their offspring for longevity studies

**DOI:** 10.1007/s11357-023-01037-4

**Published:** 2023-12-23

**Authors:** Alexandre How-Kit, Mourad Sahbatou, Lise M. Hardy, Nicolas P. Tessier, Valérie Schiavon, Hélène Le Buanec, Jean-Marc Sebaoun, Hélène Blanché, Jean-François Zagury, Jean-François Deleuze

**Affiliations:** 1 Laboratory for Genomics, Foundation Jean Dausset – CEPH, Paris, France; 2 Laboratory of Excellence GenMed, Paris, France; 3 INSERM U976 - HIPI Unit, Saint-Louis Research Institute, University of Paris, Paris, France; 4 Centre de Ressources Biologiques, Foundation Jean Dausset – CEPH, Paris, France; 5 Équipe Génomique, Bioinformatique et Chimie Moléculaire (EA 7528), Conservatoire National Des Arts et Métiers, HESAM Université, Paris, France; 6 Centre National de Recherche en Génomique Humaine, CEA, Institut François Jacob, Evry, France

**Keywords:** Cohort study, Biobanks, Biorepository, DNA, Blood samples, Aging, Longevity, Centenarians

## Abstract

The increasing aging of the human population is currently and for the coming decades a major public health issue in many countries, requiring the implementation of global public health policies promoting healthy and successful aging. Individuals are not equal in the face of aging and some can present exceptional healthspan and/or lifespan, which are notably influenced by both genetic and environmental factors. Research and studies on human aging, healthy aging and longevity should rely in particular on cohorts of long-lived individuals, also including biological samples allowing studies on the biology of aging and longevity. In this manuscript, we provide for the first time a complete description of the CEPH (Centre d’Etude du Polymophisme Humain) Aging cohort, an exceptional cohort recruited during the 90s to 2000s, including more than 1700 French long-lived individuals (≥ 90 years old) born between 1875 and 1916 as well as for some of them their siblings and offspring. Among the participants, 1265 were centenarians, including 255 semi-supercentenarians ([105–110] years old) and 25 supercentenarians (≥ 110 years old). The available anthropometric, epidemiologic and clinical data for the cohort participants are described and especially the collection of blood-derived biological samples associated with the cohort which includes DNA, cryopreserved cells and cell lines, plasma, and serum. This biological collection from the first cohort of centenarians in the world is an inestimable resource for ongoing and future molecular, cellular, and functional studies aimed at deciphering the mechanisms of human (successful) aging and longevity.

**Supplementary Information:**

The online version contains supplementary material available at 10.1007/s11357-023-01037-4.

## Introduction

Aging is defined as the progressive decline of the overall organismal fitness during adulthood, accompanied by alteration and dysfunction of several molecular, cellular and physiological functions, leading to an increased susceptibility to disease and death [1–3]. To date, the aging of the European population, including France, is one of the greatest current societal, economic and public health challenges. The demographic projections estimated in 2020 that almost a third of the European population would be aged over 65 years in 2050, nearly twice as many than in 2001 (Ageing Europe, 2020, Eurostat). It would thereby be important to be able to propose solutions to the consequences of an aging population, notably by improving healthy and successful aging. A first step toward this goal includes the comprehensive and deep understanding of the basic mechanisms of aging and longevity, which differentially affect each individual. Of note, lifespan and healthspan are closely related and some individuals can present exceptional longevity in good health compared to others [4–6]. From a holistic point of view, two paradigms have been proposed for human longevity and successful aging [7], either relying on models developed from the “compression of morbidity” hypothesis that was first stated by Fries in 1980 [8, 9], or the “deceleration aging” hypothesis [10, 11].

To date, extreme human longevity is considered to be the result of a combination of multiples factors including genetics, environment, resiliency and even chance [12]. Concerning genetics, the long-known existence of centenarian families had always suggested a genetic and heritable component of longevity [13, 14]. The heritability of human longevity, first measured in a Danish twin study [15], was estimated at between 20 and 30% [7, 16]. Moreover, the parents, siblings and offspring of long-lived individuals have a higher average lifespan than individuals without long-lived relatives [12, 16, 17]. In this context, centenarians are considered as an exceptional model for healthy aging and longevity studies, due to their remarkable ability to achieve successful aging. Thus, they escaped death or strongly debilitating diseases for most of their life and resisted well in the face of the frailties and diseases of the great age [7, 12, 16, 18, 19]. This is particularly true for genetic studies as the heritably of longevity was shown to increase with old age, notably in centenarians and (semi-)supercentenarians [20, 21].

The Center for the Study of Human Polymorphism (CEPH) was created in 1984 by Professor Jean Dausset following his Nobel Prize in Medicine in 1980, as well as by his collaborators: Professors Daniel Cohen, Howard Cann and Mark Lathrop and became in 1993 Foundation Jean Dausset – CEPH (https://www.fjd-ceph.org/accueil/historique). Its aim was to initiate and coordinate the first international collaboration to establish a genetic map of the human genome, as well as to develop genetic studies based on cohorts of patients or donors associated with a biobank of samples. In this context, the CEPH Aging cohort, also known as the CEPH Chronos or CEPH Centenarian cohort, was established in the 1990s to 2000s by the Foundation Jean Dausset – CEPH mainly to collect the blood of almost 1800 French nonagenarians, centenarians and super-centenarians as well as for some of them their offspring, in order to identify heritable genetic factors associated with longevity. This allowed the identification of the *APOE* gene [22], whose association with longevity is still the most clearly established to date as this result has been extensively replicated in other studies [23, 24]. Although largely cited in several genetic studies, the CEPH Aging cohort, which was the first cohort of centenarians in the world, has never been described in detail. In this present manuscript, we provide for the first time a complete description of the CEPH Aging cohort with all the available data from the participants as well as the collection of biological samples stored in the CEPH biobank (https://www.fjd-ceph.org/crb-du-ceph) and still available for longevity studies. We also discuss the main findings from longevity studies obtained with this cohort as well as ongoing research projects and research perspectives on this collection. This study is intended to provide to researchers of the field with a comprehensive description of the CEPH Aging cohort and its biobank, which could lay the foundations for new collaborative longevity research using cutting-edge technologies.

## Design of the CEPH Aging cohort and biobank

### Study design and participants

The CEPH Aging cohort was an observational prospective cohort study of long-lived individuals (LLIs, referred in our study as individuals reaching at least 90 years of age) and their offspring recruited between 1991 and 2002. The project recruited (i) unrelated participants being at least 99 years of age, (ii) siblings including at least two nonagenarians, as well as (iii) first-generation descendants of the sibpairs (ii). The study protocol was approved by the French regulatory authorities, including the Commission Nationale de l’Informatique et des Libertés (CNIL) (20 September 1999) for data protection and a French ethics committee “Comité Consultatif de Protection des Personnes dans la Recherche Biomedicale” (CCPPRB Paris-Saint-Antoine, approval No. 00479). All participants provided a written informed consent at the time of inclusion. The scientific project known as the Chronos Project “genetic study of longevity and senescence pathologies” and supported by the CEPH Aging cohort, was designated:

1. To locate and identify genes associated with longevity and senescence pathologies.
2. To clarify the relationship between environment and genetics in the aging process.
3. To analyze the role of these genes in longevity and associated pathologies.
4. To study aging at the cellular level.

The analysis of the clinical and biological data collected on this population of long-lived individuals correlated with the genetic data was intended to achieve the aforementioned objectives. It should lead to an understanding of the molecular and cellular mechanisms of aging and longevity and thus eventually promote new therapeutic research to improve the health status of the elderly.

### Participants

Volunteers living in metropolitan France were eligible to participate in the study if they met the criteria defined above. Relatives of an enrolled participant could also be contacted by authorized persons after the participant’s consent or by the participant himself/herself, in order to participate in the study if this relative met the family criteria defined above. Individuals not eligible to the study included minors, adults protected by law and patients in emergency situations as well as persons hospitalized without their consent, deprived of their liberty, not affiliated to a social security system and in a state of brain death.

### Follow-up schedule

An annual medical follow-up performed for the participants during at least 5 years or until their death by sending an annual health status request to the treating physicians allowed us to know the date of death of the participants. The participants and their treating physicians were informed annually by mail of the progress of the research project.

### Data collection and management

Date of birth and date of death of participants collected from volunteers, their family or clinicians were checked on civil status registers. Epidemiologic, familial, and clinical information was collected on the participants during enrollment. The information was provided in a questionnaire completed by the volunteer’s attending physician. Epidemiologic information included sociodemographic and anthropometric characteristics: sex, date and place of birth, height, weight, eye color, profession, and educational level as well as the tobacco consumption status as the sole lifestyle characteristic. Familial information included the date and place of birth of the parents, the number of siblings and the relationship between each member of a family recruited in the cohort. No racial and/or ethnical information was collected from the participants due to the French legislation. Clinical information was mainly related to cardiovascular and neurodegenerative diseases. It included pulse rate, systolic blood pressure, diastolic blood pressure, cardiovascular history (heart rhythm disorders, heart failure, high blood pressure, angina pectoris, myocardial infarction, and lower extremity arteritis), cerebrovascular history (transient ischemic attack and stroke), cognitive impairment (Parkinson’s disease, Alzheimer’s type deficit, vascular deficit), presence of osteoporosis, urinary incontinence, prostatic pathology in men, and visual impairment with the presence of cataract and macular degeneration. Various information about ongoing medical treatments was also collected at the time of inclusion. Data from the last blood test at the time of inclusion were also collected, including total, LDL and HDL cholesterol, blood glucose, urea, and creatinine levels. A Folstein mini-mental state Test (scored from 30 to 0) or a Short Portable Mental Status Questionnaire (SPMSQ, scored from 10 to 0) was also performed at the time of inclusion.

All participants were pseudonymized and their personally identifiable data, such as name and address, are kept in a separate recruitment database from other data and are not accessible to researchers. Epidemiological, clinical, and family data were anonymized and entered into the CEPH Biobase database. The genetic data that were generated in the Chronos project are also anonymous and are stored in the CEPH genetic database. These data are accessible to researchers.

### Biosampling and storage

The biological samples were collected at the time of inclusion and included the following blood-derived materials: plasma, sera, peripheral blood mononuclear cells (PBMC), and lymphoblastoid cell lines (LCL) as well as blood and LCL extracted DNA. A total of 20 ml of blood per participant were collected in vacutainer tubes, including 5 ml in dry tubes, 5 ml in heparin tubes, and 10 ml in EDTA tubes. Blood samples were processed up to 24 h after sampling. Sera and plasma were isolated after one centrifugation step at 1600 g during 10 min from dry tubes and heparin/EDTA tubes, respectively, and were stored at − 80 °C. After plasma isolation, PBMC isolated by Uni-Sep U-10 (Novamed) from blood in heparin tubes (following the supplier’s specifications) were used either for direct long term-storage or establishment of LCL by EBV infection in vitro before long term-storage. Both types of cells were stored in a 90% RPMI 1640—GlutaMAX™/FBS (90/10 v/v) and 10% DMSO medium in liquid nitrogen tanks. DNA was extracted from cell pellets isolated from blood collected in EDTA tubes and from LCL cell lines using standard phenol-chlorofom extraction method, quantified by fluorescence, analyzed by agarose gel electrophoresis for integrity assessment, and stored at − 80 °C. When possible, PBMC dry cell pellets were also isolated after centrifugation for some participants and stored at -80 °C for later RNA and protein extractions.

All samples were processed, controlled for their quality and stored at the CEPH Biobank (https://www.fjd-ceph.org/crb-du-ceph) according to the best laboratory practices and standard operating procedures put in place by the quality management system at time of collection. The CEPH Biobank was the first biobank in Europe certified in accordance with ISO 9001:2015 and ISO 20387:2018 standards in March 2020, for its collection, reception, processing, conservation and provision of biological resources of human origin. The CEPH Biobank was previously certified ISO9001 and NF S96-900 since March 2017. Storage temperatures of all samples in the cohort are monitored in real-time by the OCEAView system and software (Dickson-Oceasoft). If the minimum threshold temperature is exceeded (− 65 °C for freezers and − 171 °C for nitrogen tanks), an on-call system has been set up to safeguard samples. The use of the CEPH Biobase software developed in-house allows the traceability of each sample during its complete life cycle.

## Results

### Description of the CEPH Aging cohort

**Fig. 1:**
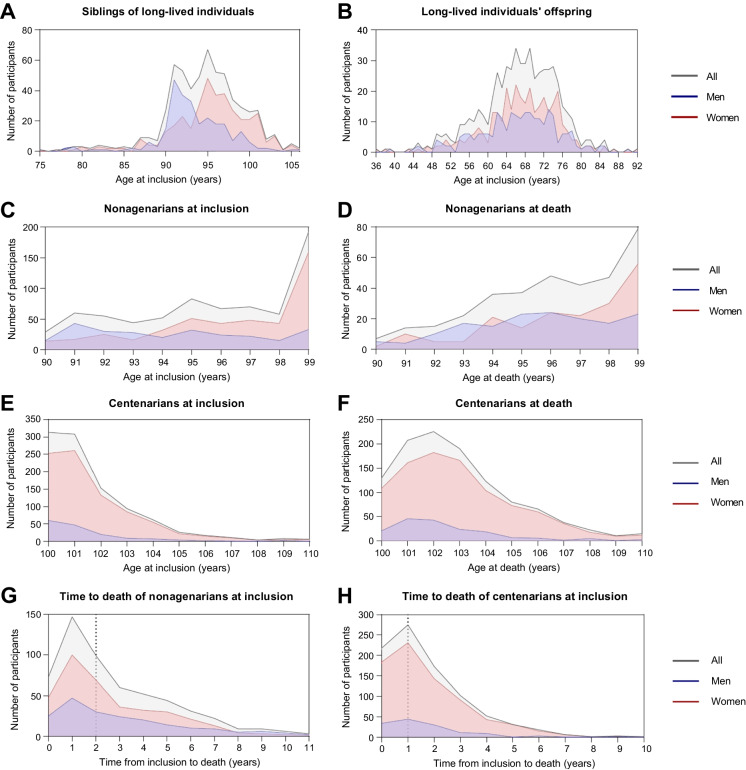
Age distribution of the CEPH Aging cohort participants, including **A** siblings of long-lived individuals with at least two nonagenarians at inclusion, **B** nonagenarians’ and centenarians’ offspring, **C** nonagenarians at inclusion and **D** at death, and **C** centenarians **E** at inclusion and **F** at death. The distribution of the delay from inclusion to death was also showed for **G** nonagenarians and **H** centenarians at inclusion as well as the median value (dotted lines). Supercentenarians were all grouped in the 110-year-old category for anonymization reasons

The overall CEPH Aging cohort included a total of 2351 French participants born for the vast majority in metropolitan France (Supplementary Fig. 1) and belonging to 1423 different families with 1 to 23 enrolled members (Table 1). The participants can be further grouped into three categories. The first included 1746 LLIs of at least 90 years of age (at inclusion, at death and/or at last known health status) born between 1875 and 1916 (Table 1). The second included 590 siblings of LLIs with at least two nonagenarians at inclusion (including 7 twin pairs) belonging to 246 families of 2 to 6 participants (Table 1). The third included 576 first-generation LLIs’ offspring in 242 families of 1 to 19 participants (Table 1). Siblings of LLIs and LLIs’ offspring were aged at inclusion between 75 to 106 (94.45 years in average) and 36 to 92 (66.7 years in average) years, respectively (Fig. 1A, B and Table 1). The cohort included more women (71%) than men, with 57.6%, 62.5%, and 75.4% of women in LLIs’ offspring, sibling of LLIs and LLIs, respectively (Table 1). Three examples of families from the CEPH Aging cohort are given in Supplementary Fig. 2.

**Table 1:** Description of the CEPH Aging cohort participants and their family relationships

	Number of participants (% of women)	Age at inclusion and sampling (years), Mean ± SD (range)	Number of different families (average, minimal, and maximal number of participants per family)
Whole cohort	2351 (71.03)	90.7 ± 14.9 (36–110^+^)	1423 (1.65; min = 1; max = 23)
Long-lived individuals^1^	1746 (75.43)	98.9 ± 4.2 (79–110^+^)	1412 (1.24; min = 1; max = 6)
Siblings of long-lived individuals^2^	590 (62.54)	94.5 ± 4.6 (75–106)	246 (2.40; min = 2; max = 6)
Long-lived individuals’ offspring^3^	576 (57.64)	66.7 ± 8.2 (36–92)	242 (2.38; min = 1; max = 19)

^1^Long-lived individuals were defined as participants with at least 90 years of age at inclusion, at death, and/or at last known health status

^2^Siblings recruited with at least two nonagenarians’ members at inclusion

^3^First generation offspring of at least one nonagenarian or centenarian parent

**Fig. 2:**
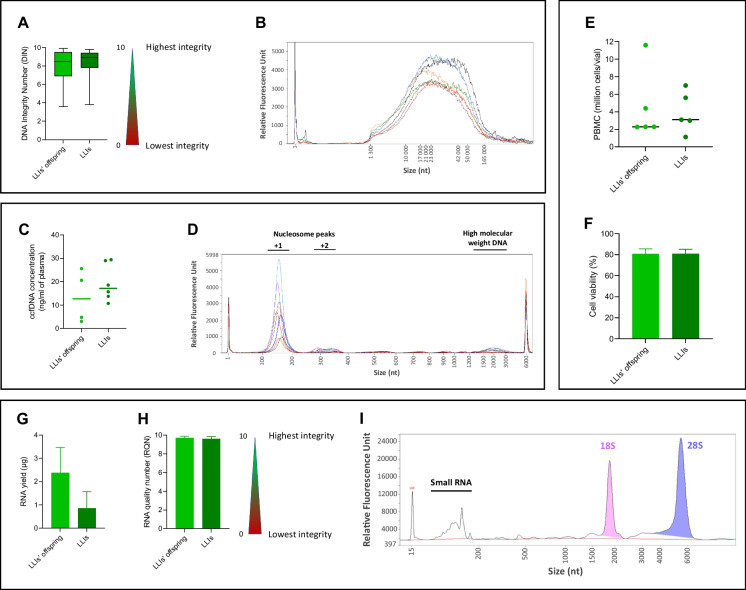
Quality control analyses of genomic DNA, plasma circulating cell-free DNA, cryopreserved PBMC and total RNA samples of the CEPH Aging cohort. **A** Integrity analysis of 169 blood extracted DNA from LLIs and LLIs’ offspring using the DNA Integrity Number (DIN) with a threshold set at 10 kb for integral DNA. **B** High-resolution Femto pulse analysis of 6 integral blood extracted DNA from LLI. **C** Fluorescence-based Qubit quantification of circulating cell-free DNA (ccfDNA) isolated from plasmas of 6 LLIs and 4 LLIs’ offspring and their **D** capillary electrophoresis analysis by Femto Pulse. Quantification **E** and viability analyses **F** of cryopreserved PBMC of 5 LLIs and 5 LLIs’ offspring by fluorescent flow cytometry. **G** Absorbance-based Nanodrop quantification of total RNA isolated from PBMCs of 3 LLIs and 3 LLIs’ offspring and their **H** integrity analyses using RNA quality number (RQN) by capillary electrophoresis on a Fragment Analyzer. **I** An example of capillary electrophoresis profile of total RNA obtained from a PBMC of an LLIs’ offspring

LLIs could be further divided into several age groups (nonagenarians (NOG), centenarians (C), semi-supercentenarians (SSC) and supercentenarians (SC) based on their age at inclusion (*n* = 1712), age at death (*n* = 1454), and age at last known health status (*n* = 1746, Fig. 1C–F and Table 2). The sex ratio of LLIs is biased toward women and increased with age, both at inclusion and at death (Fig. 2C–F and Table 2), as expected. When considering the age at death, the CEPH Aging cohort included 347 nonagenarians and 1007 centenarians of whom 218 and 15 were SSC and SC, respectively (Table 2). Of note, the delay between inclusion and death varied from 0 to 11 years for nonagenarians at inclusion and from 0 to 10 years for centenarians at inclusion, with half of the participants passing away within 2 years and 1 year after inclusion, respectively (Fig. 2G, H).

**Table 2:** Demographic characteristics of the long-lived individuals from the CEPH Aging cohort

Age range (years)	Participants (*N*) considering their age at inclusion		Participants (*N*) considering their age at death		Participants (*N*) considering their age at last known health status	
	All (% of women)	Unrelated	All (% of women)	Unrelated	All (% of women)	Unrelated
≥ 90	1712 (75.76)	1411	1454 (76.96)	1207	1746 (75.43)	1412
[90–100]; nonagenarians	710 (63.10)	487	347 (54.47)	234	481 (54.68)	321
≥ 100; centenarians	1002 (84.73)	996	1107 (84.01)	1063	1265 (83.32)	1212
[100–105]	930 (84.62)	927	874 (82.49)	841	984 (82.22)	944
[105–110]; semi-supercentenarians	66 (84.85)	66	218 (90.37)	218	256 (87.89)	255
≥ 110; supercentenarians	6 (100)	6	15 (80)	15	25 (80)	25

The anthropometric, epidemiologic, and clinical data available for the LLIs of the CEPH Aging cohort were also described in Table 3 and 4. Except for the age at inclusion, quantitative anthropometric, epidemiologic and clinical data were collected for 4.4 to 58% of the LLIs (Table 3), while categorial data on lifestyle, cardiovascular and cerebrovascular history, cognitive impairment and other diseases were collected for 17 to 73% of the LLIs (Table 4). These data showed, inter alia, a mean alteration of the consciousness in the long-lived participants with a mean Folstein score of 20.84 and a mean SPMSQ score of 6.22 (Table 3), including the presence of Parkinson’s disease and Alzheimer’s type deficit in 4% and 7% of them (Table 4), respectively. Cardiovascular history showed heart rhythm disorders, heart failure, angina pectoris, and high blood pressure in 24.6 to 39.9% of LLIs as well as an history of myocardial infarction in 7% of them (Table 4). Finally, 22%, 66%, and 71% of LLIs presented macular degeneration, cataract and visual impairment (Table 5), respectively. There was no possibility to collect racial and/or ethnical information from the participants at time of recruitment, despite its great importance for genetics studies. However, determining their racial and/or ethnical origin was feasible based on their SNP genotype data, which indicated that almost all LLI with available SNP data presented a genetic background from Europe, and more precisely from France (Supplementary Fig. 3).

**Table 3:** Quantitative epidemiologic and clinical data available for long-lived individuals from the CEPH aging cohort

	All		Men		Women	
	% of data available	Mean ± SD	% of data available	Mean ± SD	% of data available	Mean ± SD)
Anthropometry						
Age at inclusion (years)	100	98.9 ± 4.17	100	96.6 ± 4.57	100	99.64 ± 3.73
Height (cm)	55.78	160.06 ± 8.65	61.77	167.94 ± 7.1	53.83	157.11 ± 7.21
Weight (kg)	58.07	54.98 ± 12.75	62.23	65.84 ± 10.68	56.71	51.09 ± 11.08
Cardiovascular parameters						
Pulse rate (bpm)	49.94	74.23 ± 8.88	50.34	71.5 ± 8.61	49.81	75.13 ± 8.79
Systolic blood pressure (cm Hg)	58.93	13.65 ± 1.6	58.74	13.57 ± 1.45	58.99	13.67 ± 1.64
Diastolic blood pressure (cm Hg)	58.19	7.63 ± 0.87	58.04	7.66 ± 0.79	58.23	7.62 ± 0.89
Blood test						
Total cholesterol (g/L)	13.11	2.07 ± 0.55	13.75	2 ± 0.71	12.9	2.1 ± 0.49
LDL cholesterol (g/L)	4.41	1.21 ± 0.41	3.72	1.1 ± 0.43	4.63	1.23 ± 0.4
HDL cholesterol (g/L)	5.09	0.52 ± 0.19	3.96	0.52 ± 0.28	5.46	0.52 ± 0.16
Blood glucose (g/L)	30.18	1 ± 0.56	31.7	1.07 ± 0.64	29.68	0.97 ± 0.53
Urea (g/L)	20.84	0.49 ± 0.25	20.74	0.5 ± 0.18	20.88	0.49 ± 0.27
Creatinine level (mg/L)	33.56	11.06 ± 3.82	37.06	12.5 ± 3.94	32.42	10.53 ± 3.64
Cognitive function						
Folstein test (points)	20.84	19.62 ± 7.46	22.14	22 ± 6.23	20.42	18.78 ± 7.69
SPMSQ (points)	49.26	6.22 ± 3.57	51.28	7.62 ± 3.54	48.60	5.77 ± 3.47

**Table 4:** Categorical epidemiologic and clinical data available for long-lived individuals from the CEPH aging cohort

	All		Men		Women	
	% of data available	% of positive cases	% of data available	% of positive cases	% of data available	% of positive cases
Lifestyle						
Tobacco consumption^1^	72.9	10.37	76.95	33.97	71.45	3.08
Cardiovascular history						
Heart rhythm disorders	26.51	35.64	27.03	39.66	26.34	34.29
Heart failure	28.86	39.88	28.67	30.08	28.92	43.04
High blood pressure	71.93	37.10	74.59	29.69	71.07	39.64
Angina pectoris	71.07	24.58	74.12	22.64	70.08	25.24
Myocardial infarction	26.97	7.01	28.2	9.92	26.57	6.00
Lower extremity arteritis	69.07	11.53	70.62	10.56	68.56	11.85
Cerebrovascular history						
Transient ischemic attack	24.85	13.59	23.31	11.00	25.36	14.37
Stroke	26.17	7.22	26.34	6.19	26.11	7.56
Cognitive impairment						
Parkinson’s disease	24.11	4.04	22.84	7.14	24.52	3.10
Alzheimer’s type deficit	24.28	13.44	22.84	11.22	24.75	14.11
Vascular deficit	24.74	21.06	24.94	13.08	24.67	23.69
Other diseases						
Osteoporosis	58.19	45.87	62.23	16.48	56.87	56.34
Urinary problem^2^	23.19	45.19	22.61	43.30	23.38	45.78
Visual impairment^3^	17.23	71.10	14.45	19.35	15.64	20.39
Cataract	55.32	66.36	60.13	57.75	53.75	69.49
Macular degeneration	19.53	22.29	21.21	23.08	18.98	22.00

^1^Tobacco consumption includes current and former smokers

^2^Urinary problem includes prostatic pathology and/or incontinence

^3^Visual impairment includes amblyopic and blind participants

**Table 5:** Description of the biological samples available for the CEPH Aging cohort (March 2023)

Sample type	Whole cohort	LLIs	Siblings of LLIs	LLIs’ offspring
	(*n* = 2351)	(*n* = 1746)	(*n* = 590)	(*n* = 576)
	% of samples available	% of samples available	% of samples available	% of samples available
DNA	94.6	93.3	92.5	98.8
LCL origin	63.2	69.5	76.5	34
Blood origin	69.5	53.2	42.5	95.3
Cryopreserved cells	91.3	91.3	88.5	89.7
LCL	74.6	81.5	84.7	55.7
PBMC	78.2	71.5	73.9	97.9
Biofluids	96.9	92	94.2	100
Serum	75	66.8	71	99.1
Plasma	92.7	91.2	90.2	97.2

### Description of the biological collection associated with the CEPH Aging cohort

The CEPH Aging cohort included a valuable collection of blood-derived samples collected from participants at time of inclusion and stored in the CEPH biobank since the 1990s. The following is the description of the different materials still available and the assessment of their quality for ongoing and future longevity studies.

### DNA samples

DNA extracted either from LCL or blood were the most exploited biological resource from this cohort. They are still available for 94.6% participants, including 93% for LLIs and 99% for LLIs’ offspring (Table 1). While LCL DNA availability is unlimited and generally suitable for genetics studies, blood DNA samples include the genome of the whole lymphocyte population of the participants at time of sampling, and could allow for more analyses such as epigenetics studies. The total DNA aliquots of the cohort (*n* = 10,160 DNA tubes) were recently validated, ascertained or identified for their origin using our tissue-of-origin real-time PCR test [25]. The results were 98.2% concordant with the origin deduced from the CEPH sample management database that were available for 39% of the aliquots, revealing very few errors for such an old collection. Blood DNA is available for 53% of LLIs and 95% of LLI offspring from the cohort (Table 5). 990 DNA extracted from blood were assessed for integrity by capillary electrophoresis and presented a DIN (DNA Integrity Number) of 8.2 and 8.4 in average for LLIs’ offspring and LLIs, respectively, indicating the availability of blood extracted DNA with a good integrity for most participants (Fig. 2A). The DIN indicates the proportion of genomic DNA higher than the threshold that is set at 10 kb. A few DNA samples from LLIs with high DIN (> 7) were further analyzed on a Femto Pulse system to increase the size resolution. The results showed a large proportion of high molecular weight DNA over 20 kb for these samples (Fig. 2B), indicating that they should be compatible with technologies requiring high integrity DNA such as long-read third generation sequencing.

### Plasma and serum samples

Biofluid samples were available for almost 96.9% of the participants, including sera and plasmas for 75% and 92.7% of LLIs, 71% and 90.2% for sibling of LLIs, and 99.1% and 97.2% of their offspring, respectively (Table 5). Although plasmas samples were isolated after a single centrifugation step (10 min at 1600 g), we wanted to assess whether they were suitable for circulating cell-free DNA (ccfDNA) isolation and analysis, as ccfDNA might carry (epi)genomic information from multiple tissues and organs from the participants [26–28], which could be valuable for the study of aging and longevity. ccfDNA was isolated from 10 EDTA plasma samples from LLIs and LLIs’ offspring, quantified by fluorescence, and analyzed by capillary electrophoresis on the Femto Pulse System (Fig. 2C, D). ccfDNA quantification showed DNA yields of 19.5 ng and 13.5 ng per ml of plasma in average with LLIs and LLIs’ offspring, respectively (Fig. 2C), which was consistent to the previously published plasma ccfDNA quantification with this ccfDNA isolation protocol [29]. Moreover, their capillary electrophoresis profiles also showed the typical plasma ccfDNA profile, including + 1 and + 2 nucleosome peaks as well as some high molecular weight DNA (Fig. 2D). Thus, plasma samples of the cohort should be suitable to further ccfDNA analysis.

### PBMC

Cryopreserved PBMCs are an invaluable source of living biological materials from the participants of the cohort that could be used in many types of analyses, including cellular, immunophenotyping, functional, and transcriptomic studies. They were still available for 71.5% of LLIs, 73.9% of sibling of LLIs, and 97.9% of LLIs’ offspring (Table 5). To assess the quality of this material preserved since 1991 in the CEPH biobank, yield, and viability of PBMCs from 5 to 5 LLIs’ offspring were determined directly following thawing by Trypan blue exclusion test and flow cytometry using ZombieNIR. The quantity of cells on thawing varied from 1.14 to 11.6 million cells per vial (4.3 million cells on average, Fig. 2E) and viable cells were measured on average at 80.8% and 80.7% for samples from LLIs and LLIs’ offspring, respectively (Fig. 2F), indicating an important amount of well-preserved and viable cells that should be suitable for downstream analyses (Fig. 2E, F). We also evaluated the quantity and quality of RNA isolated from PBMCs from 3 to 3 LLIs’ offspring. An average of 1.6 µg of RNA of high quality (average RQN = 9.65) was isolated from these cells (Fig. 2G–I), which should be suitable for transcriptomic analyses.

### Lymphoblastoid cell lines

LCL provide an unlimited source of DNA from the participants of the cohort and could also be used in cellular, functional and pharmacogenomics studies in vitro [30, 31]. They were available for 81.5% of LLIs, 84.7% of siblings of LLIs, and 55.7% of LLIs’ offspring, respectively (Table 5).

## Discussion

Population aging is a major concern affecting many countries, today and in the decades to come, since 16% of the world population will be over age 65 in 2050, including 459 million people over 80 years old (UN World Population Prospects 2022). Research on aging, healthy aging and longevity is thereby becoming important due to this global public health issue facing most countries and should in part be based on cohorts of long-lived individuals, including those with the most extreme phenotype. The CEPH Aging cohort described in our manuscript was the first cohort of centenarians in the world including a biobank of blood-derived samples. It enrolled more than a thousand French long-lived participants from 1991 to 2002, including 1265 centenarians born between 1875 and 1916 as well as their siblings (*n* = 588 siblings) and first-generation offspring (*n* = 576, Table 2). The objective of this cohort was to provide researchers biological samples as well as epidemiologic and clinical data from the participants in order to conduct research at the level of molecular and cellular biology of aging and longevity at a time when very few cohorts of LLIs with such available biological material were established. We have listed all the different genetic, biochemical and cell biology studies based on the CEPH Aging cohort and summarized their findings in Supplementary Table 1, which are also described below.

The Chronos project was the first study on this cohort to look at the genetics of longevity and pathologies of senescence. It allowed the identification of APOE (*p* < 0.001) and ACE (*p* < 0.01) as the first genes associated with human longevity in 1994 using a candidate gene approach in a case–control study including 338 centenarians and 410 adults aged 20–70 years [22]. However, while *APOE* association was largely replicated in many other studies [23, 24], ACE association should be considered as a false positive signal due to genotyping issues [32]. Other genetic investigations on this cohort identified the loss of lymphocytes’ telomeric DNA during aging and in centenarians [33], sex-dependent HLA-DR alleles associated with individual and familial longevity using 533 centenarians and 163 nonagenarian siblings [34], and a lack of association of C282Y mutation in the *HFE* gene, as well as drug-metabolizing enzyme-coding genes *NAT2*, *GSTM1*, and *CYP2D,* with human longevity [35, 36]. Genetic data from the cohort also allowed the development and assessment of mathematical models for the study of genetics of aging and longevity [37, 38]. In addition, centenarians of the CEPH aging cohort were also included in several European genetic association studies, mainly as a French population for validation/replication experiments and meta-analysis studies [39–49]. Thus, SNP genotype data from the cohort allowed the replication of associations with longevity of rs2706372 (*p* = 2.69 × 10^−3^) that is located in a region encompassing *RAD50* and *IL13* [42], as well as rs4946935 (*p* = 0.022) and rs12206094 (*p* = 0.008) that are located in the *FOXO3* gene region [44]. Moreover, meta-analysis studies have also identified associations of rs2149954, located on chromosome 5q33.3, with longevity (*p* = 1.74 × 10^−8^) and survival beyond 90 years of age (*p* = 0.003) [41], and of rs7676745 near *GPR78* with longevity (*p* = 4.3 × 10^−8^) [43].

In addition to these genetic studies, the biological samples from CEPH Aging cohort were used in a few biochemical, cellular and immunophenotyping studies (Supplementary Table 1). Biochemical studies determined the concentration of few proteins and metabolites and identified high levels of homocysteine [50], lipoprotein(a) [51], and apolipoprotein(a) [51] and low levels of folate [50] in plasmas of centenarians compared to younger controls, and also measured the enzymatic activity of ACE in centenarians’ sera [52]. Cellular and immunophenotyping studies were based on a small number of markers and described CD28^+^ T cell decline that preferentially affected CD8^+^ T cells in centenarians compared to younger controls [53, 54]. More recently, we have published the first epigenetic study on this cohort showing that centenarians (*n* = 214) and LLIs’ offspring (*n* = 143) presented lower epigenetic age than their chronological age with four epigenetic clocks based on a small number of CpGs compared to individuals from the French general population (*n* = 143) [55], suggesting that epigenetic aging could potentially be decelerated in these individuals.

The quantity and quality of the well-preserved biological material still available for this cohort might allow many research perspectives. Ongoing studies and projects on this cohort include the Mitochongevity and Agenomics projects. Mitochongevity (https://www.france-genomique.org/projet/mitochongevity/), started in 2022, aims to analyze the mitochondrial genome (mtDNA) and methylome of centenarians and LLIs’ offspring by long-read ultra-deep third generation sequencing and identify germline and somatic (epi)genetic variations of mtDNA associated with human longevity as well as their transgenerational inheritance. In addition, the Agenomics project, started in 2023, aims at exploiting genomic data from centenarians of the CEPH Aging cohort and longitudinal cohorts for the development of new strategies targeting aging. Agenomics will notably perform whole genome sequencing of more than 1000 centenarians from the cohort by short-read next-generation sequencing and is also intended to develop a European consortium to study longevity using multi-omics approaches and cohorts of long-lived individuals. Other research avenues on the biological samples of this cohort might include methylation analyses from blood extracted DNA, transcriptomic and immuno-phenotyping analyses of the cryopreserved immune cells, and the analysis of metabolites, proteins, and circulating cell-free nucleic acids from plasma and serum, using all available state-of-the-art technologies. We can also speculate about some clinical applications which could arise from the findings on this cohort. Cellular and molecular signals and signatures identified in centenarians could thereby present a great potential as biomarkers of healthy aging and longevity in the general population, especially in a context of P4 medicine. More specifically, positive genetic associations identified could be combined to enable the construction of a polygenic risk score for healthy aging and longevity, notably in the French population. Moreover, potential bio-active molecules identified in centenarians from the cohort, for example circulating miRNA enclosed in micro-vesicles and exosomes, could also present some longevity properties, which might allow their use in anti-aging or healthy aging therapies.

In summary, we provided for the first time a complete description of the CEPH Aging cohort including the biological samples still available to date for ongoing and future longevity studies. We acknowledge that the cohort presents some limitations, including the low amount of epidemiologic and clinical data collected from the participants (from 4 to 76%, Table 3 and 4) that cannot be completed, the lack of longitudinal data except death data and the absence of samples non-derived from blood. Of note, being able to stratify centenarians based on their health status would be important for longevity studies, as they present a high degree of health heterogeneity that can modulate their survival prospects [56]. This might also affect the identification of biomarkers and/or mechanisms of longevity, as exemplified in studies on circulating cell-free nucleic acids from healthy and unhealthy centenarians [28]. However, the CEPH Aging cohort also presents several strengths that lie in (i) the extreme longevity phenotype for an important number of participants (1265 centenarians, including 256 SSC and 25 SC, Table 2), (ii) the period of birth of the long-lived participants (1875–1916) that corresponded to a time when very few individuals could expect to achieve such longevity, and (iii) the high amount of well-preserved biological samples from most participants that is still available for many downstream experimental studies. Thus, this ancient collection could be more valuable than ever for many longevity studies in a context of global aging of the European and world populations, especially since the development of new technologies in the field of genomics.

## Methods

### Blood extracted gDNA analysis

A total of 990 blood extracted genomic DNA were analyzed for integrity by calculating their DNA Integrity Number (DIN, threshold set at 10 kb) using Genomic DNA ScreenTape Analysis on a 4200 TapeStation System (Agilent) according to the manufacturer’s instructions. Some high molecular weight genomic DNA samples were further analyzed by highly sensitive high-resolution capillary electrophoresis using the Genomic DNA 165-kb Kit on a Femto Pulse System (Agilent) according to the manufacturer’s instructions.

### Plasma ccfDNA analysis

A total of 10 EDTA plasma samples (1 ml) were thawed on ice and tested with Quantofix EDTA 0-400MG/L strips (Macherey–Nagel) to confirm the anticoagulant used. Then, ccfDNA was isolated from plasma using the QIAamp Circulating Nucleic Acid Kit (Qiagen) according to manufacturer’s instructions and quantified with the Qubit HS dsDNA Kit (Life Technologies). ccfDNA was further analyzed by highly sensitive high-resolution capillary electrophoresis using the Ultra Sensitivity NGS Kit on a Femto Pulse System (Agilent) according to manufacturer’s instructions.

### PBMC RNA extraction and analysis

Cryopreserved PBMCs were thawed during 1 min in a water bath at 37 °C, then 1 ml of PBMC was transferred into 9 ml of PBS 1 × and pelleted during 10 min at 2000 RPM. Total RNA was isolated from the pelleted cells using miRNeasy Mini Kit (QIAGEN, Germany) according to manufacturer’s instructions. RNA concentrations were measured with a NanoDrop 2000c spectrophotometer (Thermo Scientific, France) and the RNA integrity was assessed using HS RNA Kit on a Fragment Analyzer (Agilent Technologies, France) according to manufacturer’s instructions.

### PBMC viability analysis

Cryopreserved PBMC were thawed during 1 min in a water bath at 37 °C, then 1 ml of PBMC was transferred into 9 ml of RPMI media and incubated at 37 °C until viability analyses. Cell quantification and viability analyses were performed using PBMC from LLIs and LLIs’ offspring by Trypan blue exclusion test and by flow cytometry on an CYTEK Aurora cytometer (Cytek Bioscience) with ZombieNIR viability dye staining.

### Supplementary Information

Below is the link to the electronic supplementary material.

Supplementary file1 (PDF 577 KB)

## Data Availability

Data sharing is not applicable to this article as no new genetic or genomic data were created or analyzed in this study.
