# Correction to: The CEPH aging cohort and biobank: a valuable collection of biological samples from exceptionally long‑lived French individuals and their offspring for longevity studies

**DOI:** 10.1007/s11357-024-01085-4

**Published:** 2024-01-26

**Authors:** Alexandre How‑Kit, Mourad Sahbatou, Lise M. Hardy, Nicolas P. Tessier, Valérie Schiavon, Hélène Le Buanec, Jean‑Marc Sebaoun, Hélène Blanché, Jean‑François Zagury, Jean‑François Deleuze

**Affiliations:** 1 Laboratory for Genomics, Foundation Jean Dausset – CEPH, Paris, France; 2 Laboratory of Excellence GenMed, Paris, France; 3 INSERM U976 ‑ HIPI Unit, Saint-Louis Research Institute, University of Paris, Paris, France; 4 Centre de Ressources Biologiques, Foundation Jean Dausset – CEPH, Paris, France; 5 Équipe Génomique, Bioinformatique et Chimie Moléculaire (EA 7528), Conservatoire National Des Arts et Métiers, HESAM Université, Paris, France; 6 Centre National de Recherche en Génomique Humaine, CEA, Institut François Jacob, Evry, France

**Correction to: GeroScience**

10.1007/s11357-023-01037-4

The original version of this article contained a few errors introduced during the typesetting process.

There were errors in the brackets used to delimit age intervals in the abstract and Table 2. We have corrected the age intervals to avoid any confusion.

“semi-supercentenarians ([105–110] years old)” has been corrected to “semi-supercentenarians ([105–109] years old)” in the Abstract.

“[90–100]; nonagenarians”, “[100–105]”, and “[105–110]; semi-supercentenarians” have been corrected to “[90–99]; nonagenarians”, “[100–104]”, and “[105–109]; semi-supercentenarians” in Table 2, respectively. 

New Table 2:

**Table 2:** Demographic characteristics of the long-lived individuals from the CEPH Aging cohort

Age range (years)	Participants (N) considering their age at inclusion		Participants (N) considering their age at death		Participants (N) considering their age at last known health status	
	All (% of women)	Unrelated	All (% of women)	Unrelated	All (% of women)	Unrelated
≥ 90	1712 (75.76)	1411	1454 (76.96)	1207	1746 (75.43)	1412
[90-99] ; nonagenarians	710 (63.10)	487	347 (54.47)	234	481 (54.68)	321
≥100 ; centenarians	1002 (84.73)	996	1107 (84.01)	1063	1265 (83.32)	1212
[100-104]	930 (84.62)	927	874 (82.49)	841	984 (82.22)	944
[105-109] ; semi-supercentenarians	66 (84.85)	66	218 (90.37)	218	256 (87.89)	255
≥110 ; supercentenarians	6 (100)	6	15 (80)	15	25 (80)	25

Table 3 was incorrectly justified and had a typo, which has been corrected.

New Table 3:

**Table 3:** Quantitative epidemiologic and clinical data available for long-lived individuals from the CEPH Aging cohort

	**All**		**Men**		**Women**	
	**% of data available**	**Mean ± SD**	**% of data available**	**Mean ± SD**	**% of data available**	**Mean ± SD**
**Anthropometry**						
Age at inclusion (years)	100	98.9 ± 4.17	100	96.6 ± 4.57	100	99.64 ± 3.73
Height (cm)	55.78	160.06 ± 8.65	61.77	167.94 ± 7.1	53.83	157.11 ± 7.21
Weight (kg)	58.07	54.98 ± 12.75	62.23	65.84 ± 10.68	56.71	51.09 ± 11.08
**Cardiovascular parameters**						
Pulse rate (bpm)	49.94	74.23 ± 8.88	50.34	71.5 ± 8.61	49.81	75.13 ± 8.79
Systolic blood pressure (cm Hg)	58.93	13.65 ± 1.6	58.74	13.57 ± 1.45	58.99	13.67 ± 1.64
Diastolic blood pressure (cm Hg)	58.19	7.63 ± 0.87	58.04	7.66 ± 0.79	58.23	7.62 ± 0.89
**Blood test**						
Total cholesterol (g/L)	13.11	2.07 ± 0.55	13.75	2 ± 0.71	12.9	2.1 ± 0.49
LDL cholesterol (g/L)	4.41	1.21 ± 0.41	3.72	1.1 ± 0.43	4.63	1.23 ± 0.4
HDL cholesterol (g/L)	5.09	0.52 ± 0.19	3.96	0.52 ± 0.28	5.46	0.52 ± 0.16
Blood glucose (g/L)	30.18	1 ± 0.56	31.7	1.07 ± 0.64	29.68	0.97 ± 0.53
Urea (g/L)	20.84	0.49 ± 0.25	20.74	0.5 ± 0.18	20.88	0.49 ± 0.27
Creatinine level (mg/L)	33.56	11.06 ± 3.82	37.06	12.5 ± 3.94	32.42	10.53 ± 3.64
**Cognitive function**						
Folstein test (points)	20.84	19.62 ± 7.46	22.14	22 ± 6.23	20.42	18.78 ± 7.69
SPMSQ (points)	49.26	6.22 ± 3.57	51.28	7.62 ± 3.54	48.60	5.77 ± 3.47

We have also corrected gene names that were not italicized in the Discussion.

 “It allowed the identification of APOE (*p* < 0.001) and ACE (*p* < 0.01) as the first genes associated with human longevity in 1994 using a candidate gene approach in a case–control study including 338 centenarians and 410 adults aged 20–70 years [22]. However, while *APOE* association was largely replicated in many other studies [23, 24], ACE association should be considered as a false positive signal due to genotyping issues [32].” was corrected to “It allowed the identification of *APOE* (*p* < 0.001) and *ACE* (*p* < 0.01) as the first genes associated with human longevity in 1994 using a candidate gene approach in a case–control study including 338 centenarians and 410 adults aged 20–70 years [22]. However, while *APOE* association was largely replicated in many other studies [23, 24], *ACE* association should be considered as a false positive signal due to genotyping issues [32].”

