# Supplementary material for: The CEPH aging cohort and biobank: a valuable collection of biological samples from exceptionally long-lived French individuals and their offspring for longevity studies
Source: GeroScience. 2023 Dec 23;46(2):2681–95. doi: 10.1007/s11357-023-01037-4 (PMC10828222; doi:10.1007/s11357-023-01037-4)
Supplement: Supplementary file 1 — Supplementary file1 (PDF 577 KB) [file 11357_2023_1037_MOESM1_ESM.pdf]

Supplementary Information for: The CEPH Aging cohort and biobank: a valuable collection of biological samples from exceptionally long-lived French individuals and their offspring for longevity studies

Alexandre How-Kit[1,2ǂ], Mourad Sahbatou[1], Lise M. Hardy[1,2], Nicolas P. Tessier[1], Valérie Schiavon[3], Hélène Le Buanec[3], Jean-Marc Sebaoun[3], Hélène Blanché[2,3], Jean-François Zagury[5] & Jean-François Deleuze[1,2,3,6ǂ]

[1] Laboratory for Genomics, Foundation Jean Dausset – CEPH, Paris, France

[2] Laboratory of Excellence GenMed, Paris, France

[3] Centre de Ressources Biologiques, Foundation Jean Dausset – CEPH, Paris, France

[4] Saint-Louis Research Institute, INSERM U976 - HIPI Unit, University of Paris, Paris, France

[5] Équipe Génomique, Bioinformatique et Chimie Moléculaire (EA 7528), Conservatoire National des Arts et Métiers, HESAM Université, Paris, France.

[6] Centre National de Recherche en Génomique Humaine, CEA, Institut François Jacob, Evry, France

[ǂ] ***Correspondence to:***

Alexandre How-Kit, Ph.D., Laboratory for Genomics, Foundation Jean Dausset - CEPH, Paris, F-75010, France, Tel.: +33-(0)1- 53725146, email: alexandre.how-kit@fjd-ceph.org

Jean-François Deleuze, Ph.D., Foundation Jean Dausset - CEPH, Paris, F-75010, France, Tel.: +33-(0)1-53725120, email: deleuze@cnrgh.fr

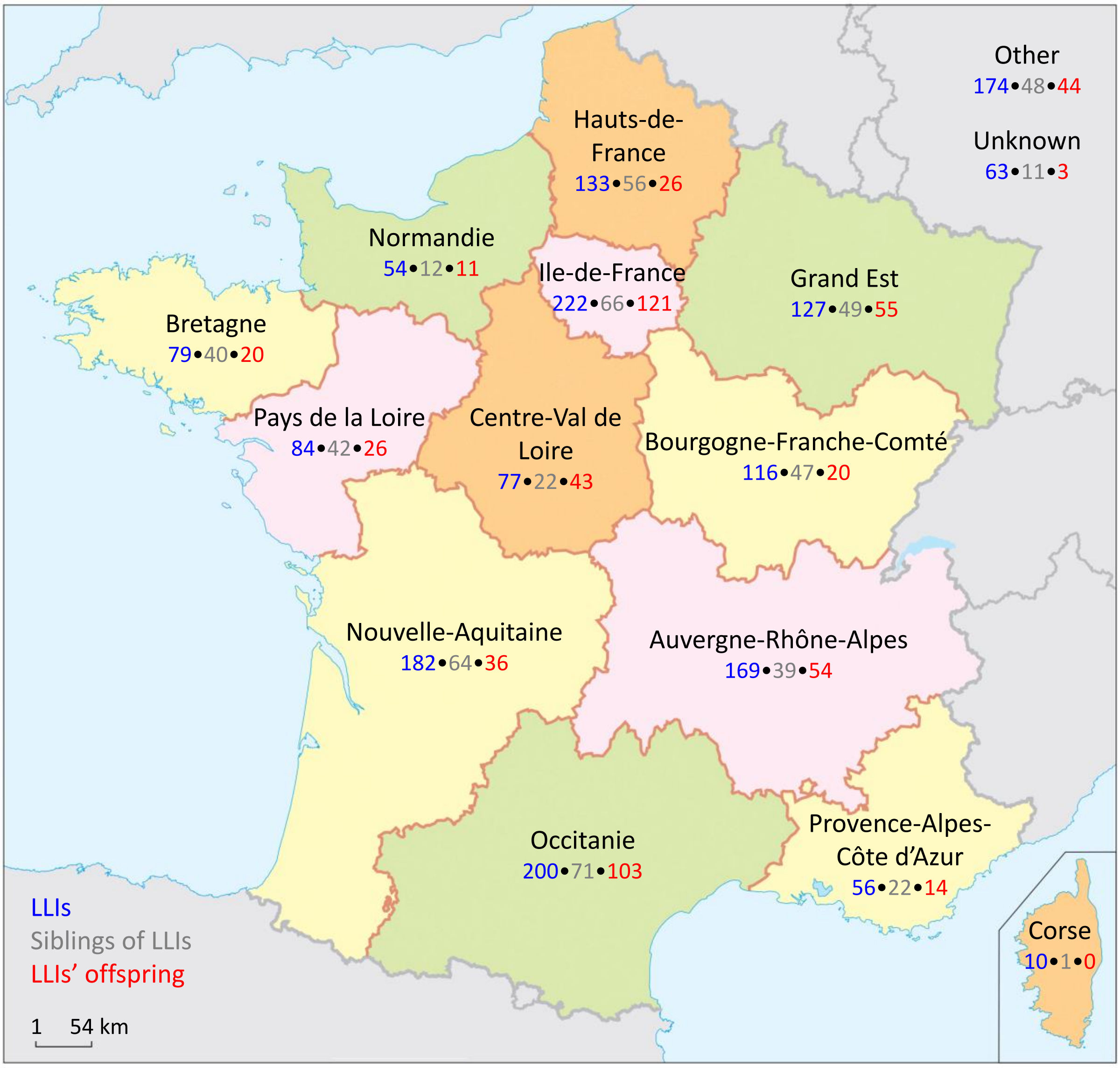

Supplementary Figure 1: Distribution of birthplace of CEPH Aging cohort participants by the 13 French metropolitan regions. The figure was modified from a free license image from IGN (National Institute of Geographic and Forest Information).

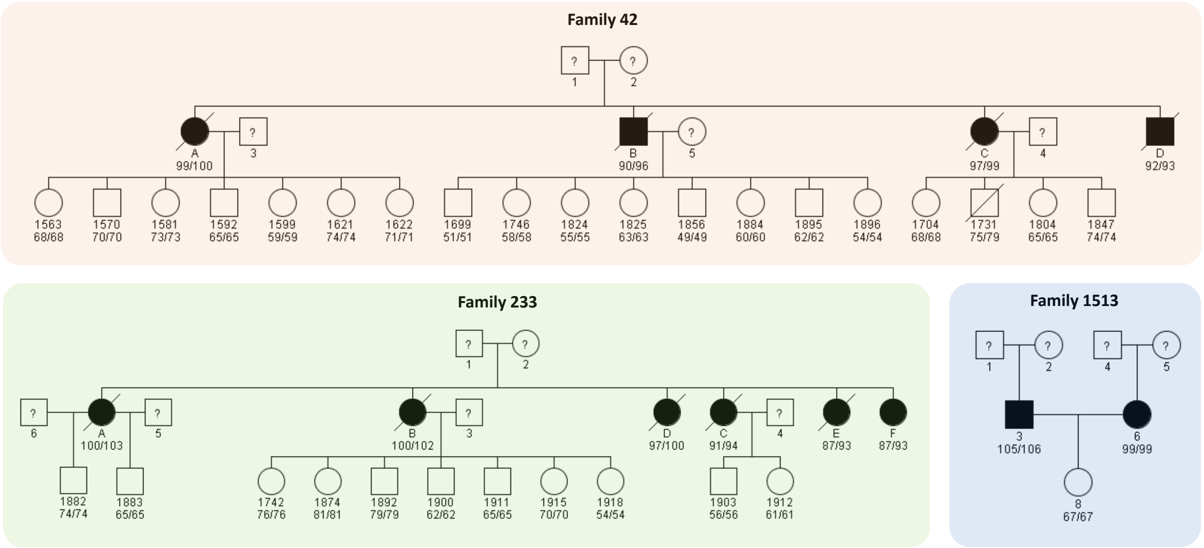

Supplementary Figure 2: Examples of three families of long-lived individuals and their offspring included in the CEPH Aging cohort. The age at inclusion and at last known health status of the participants is given at the below left and below right of each individual's identifier, respectively. Deceased cohort participants are crossed out. Long-lived participants are colored black. "?" designates individuals not participating in the cohort.

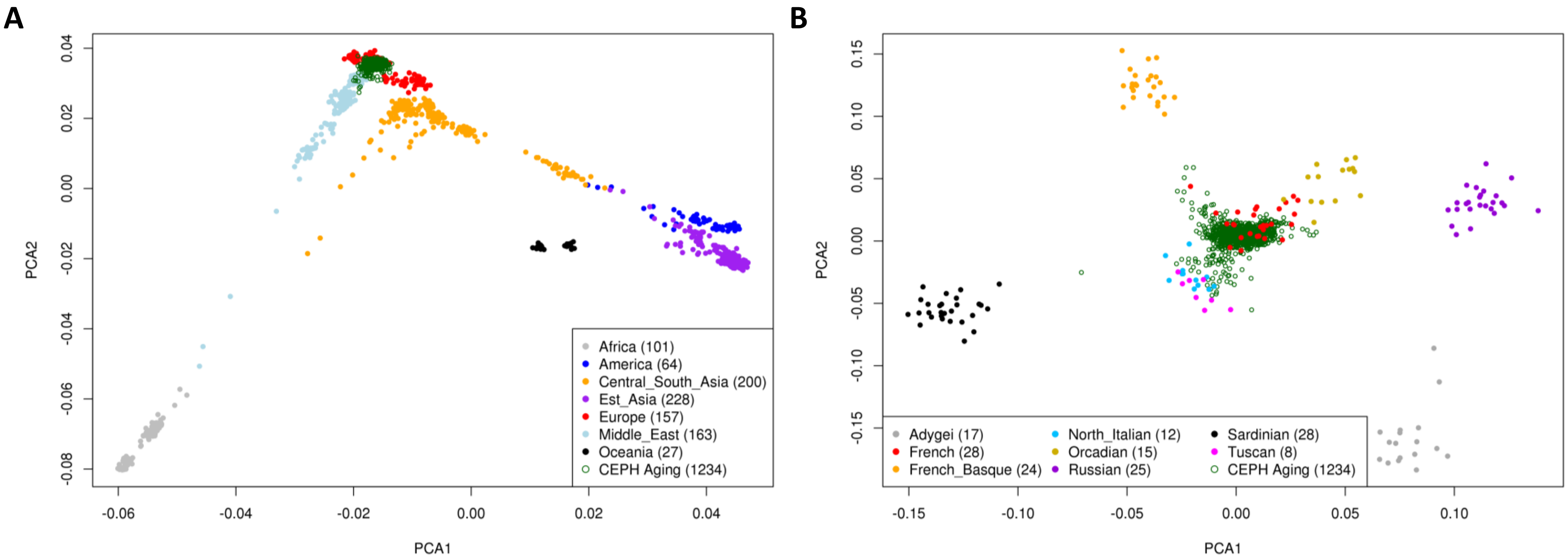

Supplementary Figure 3: Genetic diversity of 1234 LLI from the CEPH Aging cohort among Human populations. Principal component analysis (PCA) was performed using SNA data either from (**A**) all or (**B**) only European populations of the CEPH-HGDP (Human Genome Diversity Cell Line Project [1, 2]) panel. A subset of SNP markers (n=42 856) that are in very reduced linkage disequilibrium (LD) with each other was used in order to perform PCA based on the variance-standardized relationship matrix implemented in plink software. The LD pruning is obtained using Plink's "indep-pairwise" option with the following parameters: sliding window size of 500 SNPs, overlapping by 50 and pairwise R^2 threshold is 0.1. We have based the principal components on a subset of HGDP unrelated samples (940 individuals worldwide and 157 Europeans individuals) and then projected the SNP data from 1234 LLI [3] from the CEPH Aging onto these PCs.

1. Cann, H.M., et al., *A human genome diversity cell line panel.* Science, 2002. **296**(5566): p. 261-2.
2. Li, J.Z., et al., *Worldwide human relationships inferred from genome-wide patterns of variation.* Science, 2008. **319**(5866): p. 1100-4.
3. Deelen, J., et al., *A meta-analysis of genome-wide association studies identifies multiple longevity genes.* Nat Commun, 2019. **10**(1): p. 3669.

Supplementary Table 1: List of all studies and publications based on the CEPH Aging cohort

Authors	Title	Journal	Year	Issue(Volume): Pages	Research Domain	Article Types	Samples from the CEPH aging cohort analyzed	Findings on the CEPH aging cohort
Schächter F, Cohen D, Kirkwood T	Prospects for the genetics of human longevity	Hum Genet	1993	91(6):519-26	Genetics	Review	-	-
Vaziri H, Schächter F, Uchida I, Wei L, Zhu X, Effros R, Cohen D, Harley CB	Loss of telomeric DNA during aging of normal and trisomy 21 human lymphocytes	Am J Hum Genet	1993	52(4):661-7	Genetics	Research Article	18 individuals aged over 99 years old	Centenarians presented a mean telomeric "terminal restriction fragment" length from PBMC of 5.28 ± 0.4 kpb
Schächter F, Faure-Delanef L, Guénot F, Rouger H, Froguel P, Lesueur-Ginot L, Cohen D	Genetic associations with human longevity at the APOE and ACE loci	Nat Genet	1994	6(1):29-32	Genetics	Research Article	338 individuals aged over 99 years old	Association of APOE (*p*<0.001) and ACE (p<0.01) with longevity
Effros RB, Boucher N, Porter V, Zhu X, Spaulding C, Walford RL, Kronenberg M, Cohen D, Schächter F	Decline in CD28+ T cells in centenarians and in long-term T cell cultures: a possible cause for both in vivo and in vitro immunosenescence	Exp Gerontol	1994	29(6):601-9	Cell Biology	Research Article	28 healthy centenarians (>99 years old)	Decrease in the % of $CD28^+$ T cell correlated to CD4/CD8 ratio decrease in centenarians (r^2 = 0.695, *p*<0.0001)
Schächter F, Boucher N, Lesueur-Ginot L, Dufour F, Lethrosne F, Dufeu-Duchesne T, Le Coniat M, Berger R, Cohen D	[Cellular senescence and survival of T lymphocytes]	C R Acad Sci III	1995	318(5):563-72	Cell Biology	Research Article	Full article not found	Full article not found
Faure-Delanef L, Quéré I, Zouali H, Cohen D	Human longevity and R506Q factor V gene mutation	Thromb Haemost	1997	78(3):1160	Genetics	Research Article	Full article not found	Full article not found
Faure-Delanef L, Quéré I, Chassé JF, Guerassimenko O, Lesaulnier M, Bellet H, Zittoun J, Kamoun P, Cohen D	Methylenetetrahydrofolate reductase thermolabile variant and human longevity	Am J Hum Genet	1997	60(4):999-1001	Genetics	Research Article	458 centenarians (>99 years old) and 106 nonagenarians	Lower frequency of +/+ MTHFR genotypes (C677T) in centenarians and nonagenarians, although not significant
Ivanova R, Lepage V, Charron D, Schächter F	Mitochondrial genotype associated with French Caucasian centenarians	Gerontology	1998	44(6):349	Genetics	Research Article	248 centenarians (>99 years old)	Association of a variant at position 9055 of mtDNA ATP6 gene with longevity (*p*<0.01)
Muiras ML, Verasdonck P, Cottet F, Schächter F	Lack of association between human longevity and genetic polymorphisms in drug-metabolizing enzymes at the NAT2, GSTM1 and CYP2D6 loci	Hum Genet	1998	102(5):526-32	Genetics	Research Article	552 centenarians (>99 years old)	No significant difference was found at *CYP2D6*, *NAT2*, and *GSTM1* loci (6 variants testes) between centenarians and controls
Boucher N, Dufeu-Duchesne T, Vicaut E, Farge D, Effros RB, Schächter F	CD28 expression in T cell aging and human longevity	Exp Gerontol	1998	33(3):267-82	Cell Biology	Research Article	97 centenarians (>99 years old)	Significant decline in CD28+ T cells (*p*<0.0001) in centenarians, and preferentially CD8+ subset
Muiras ML, Müller M, Schächter F, Bürkle A	Increased poly(ADP-ribose) polymerase activity in lymphoblastoid cell lines from centenarians	J Mol Med	1998	76(5):346-54	Cell Biology	Research Article	49 centenarians (>99 years old)	Maximal poly(ADP-ribose) polymerase activity in lymphoblastoid cell lines was significantly higher in centenarians than in controls (*p* = 0.0031)
Ivanova R, Hénon N, Lepage V, Charron D, Vicaut E, Schächter F	HLA-DR alleles display sex-dependent effects on survival and discriminate between individual and familial longevity	Hum Mol Genet	1998	7(2):187-94	Genetics	Research Article	533 centenarians and 163 nonagenarian siblings	DR7, DR11 and DR13 alleles are higher in frequency in longevous males, in women from longevous siblings and in centenarians, respectively

Faure-Delanef L, Baudin B, Bénéteau-Burnat B, Beaudoin JC, Giboudeau J, Cohen D	Plasma concentration, kinetic constants, and gene polymorphism of angiotensin I-converting enzyme in centenarians	Clin Chem	1998	44(10):2083-7	Genetics/Biochemistry	Research Article	394/150 centenarians (>99 years old) for genetics/biochemical analysis, respectively	*ACE* D allele and *ACE* D/D genotype were more frequent in centenarians. I/D polymorphism was correlated with circulating ACE activity in centenarians
Thillet J, Doucet C, Chapman J, Herbeth B, Cohen D, Faure-Delanef L	Elevated lipoprotein(a) levels and small apo(a) isoforms are compatible with longevity: evidence from a large population of French centenarians	Atherosclerosis	1998	136(2):389-94	Biochemistry	Research Article	109 centenarians (>99 years old)	Lp(a) levels were higher in centenarians than in the normolipidemic control group ($p<0.005$). The distribution of apo(a) isoforms was significantly shifted towards small isoform size in the centenarian population (p = 0.04).
Toupance B, Godelle B, Gouyon PH, Schächter F	A model for antagonistic pleiotropic gene action for mortality and advanced age	Am J Hum Genet	1998	62(6):1525-34	Genetics	Research Article	Reuse of ACE genotypic data from centenarians	Development of a model for antagonistic pleiotropic gene action for mortality and advanced age
Cottet F, Blanché H, Verasdonck P, Le Gall I, Schächter F, Bürkle A, Muiras ML	New polymorphisms in the human poly(ADP-ribose) polymerase-1 coding sequence: lack of association with longevity or with increased cellular poly(ADP-ribosyl)ation capacity	J Mol Med	2000	78(8):431-40	Genetics	Research Article	324 centenarians (>99 years old)	No significant enrichment of any of the four PARP-1 polymorphisms tested in centenarians versus controls
Némani M, Sahbatou M, Blanché H, Thomas G, Pascoe L	The efficiency of genetic analysis of DNA from aged siblings to detect chromosomal regions implicated in premature mortality and ageing	Mech Aging Dev	2000	119 (1-2):25-39	Genetics	Research Article	188 sibships of nonagenarians and centenarians (n=434) and their children (n=124)	No excess sharing of alleles among 7 polymorphisms near *APOE* was detected in aged siblings
Blanché H, Cabanne L, Sahbatou M, Thomas G	A study of French centenarians: Are ACE and APOE associated with longevity?	C R Acad Sci III	2001	324 :129-135	Genetics	Research Article	560 centenarians (mean actual age or age at death = 103.1 years)	Replication of *APOE* (but not *ACE*) alleles ($p = 1.2x10^{-8}$) and genotypes (p = 3.4x10-8) with longevity in extended cohort.
Busson-Le Coniat M, Boucher N, Blanché H, Thomas G, Berger R	Chromosome studies of in vitro senescent lymphocytes: nonrandom trisomy 2	Ann Genet	2002	45(4):193-6	Cell Biology	Research Article	3 centenarians	Identification of chromosomal abnormalities in lymphocytes cultured *in vitro* from centenarians (1/3)
Geesaman BJ, Benson E, Brewster SJ, Kunkel LM, Blanché H, Thomas G, Perls TT, Daly MJ, Puca AA	Haplotype-based identification of a microsomal transfer protein marker associated with the human lifespan	Proc Natl Acad Sci U S A	2003	100(24):14115-20	Genetics	Research Article	564 centenarians (>99 years old)	No replication of the association of microsomal transfer protein marker and longevity in the CEPH Aging cohort
Coppin H, Bensaid M, Fruchon S, Borot N, Blanché H, Roth MP	Longevity and carrying the C282Y mutation for haemochromatosis on the HFE gene: case control study of 492 French centenarians	BMJ	2003	327(7407):132-3	Genetics	Research Article	492 centenarians (>99 years old)	No association of C282Y mutation for haemochromatosis on the *HFE* gene with longevity
Flachsbart F, Caliebe A, Kleindorp R, Blanché H, von Eller-Eberstein H, Nikolaus S, Schreiber S, Nebel A	Association of FOXO3A variation with human longevity confirmed in German centenarians	Proc Natl Acad Sci U S A	2009	106(8):2700-5	Genetics	Research Article	535 centenarians (>99 years old)	No replication of the association of 3 *FOXO3A* SNP with longevity

Nebel A, Flachsbart F, Till A, Caliebe A, Blanché H, Arlt A, Häsler R, Jacobs G, Kleindorp R, Franke A, Shen B, Nikolaus S, Krawczak M, Rosenstiel P, Schreiber S	A functional EXO1 promoter variant is associated with prolonged life expectancy in centenarians	Mech Ageing Dev	2009	130(10):691-9	Genetics	Research Article	455 female centenarians	Replication of the longevity association of *EXO1* promoter SNP rs1776180 (p=0.0441, ajusted for *APOE*)
Lescai F, Blanché H, Nebel A, Beekman M, Sahbatou M, Flachsbart F, Slagboom E, Schreiber S, Sorbi S, Passarino G, Franceschi C.	Human longevity and 11p15.5: a study in 1321 centenarians.	Eur J Hum Genet.	2009	17(11):1515-9	Genetics	Research Article	546 centenarians (>99 years old)	No replication of the association of 8 *SIRT3* SNP with longevity
Caliebe A, Kleindorp R, Blanché H, Christiansen L, Puca AA, Rea IM, Slagboom E, Flachsbart F, Christensen K, Rimbach G, Schreiber S, Nebel A	No or only population-specific effect of PON1 on human longevity: a comprehensive meta-analysis	Ageing Res Rev	2010	9(3):238-44	Genetics	Research Article/Meta-Analysis	541 centenarians (>99 years old)	No significant association of *PON1* 192 Q/R with longevity
Flachsbart F, Franke A, Kleindorp R, Caliebe A, Blanché H, Schreiber S, Nebel A	Investigation of genetic susceptibility factors for human longevity - A targeted nonsynonymous SNP study	Mutat Res	2010	694(1-2):13-9	Genetics	Research Article	541 centenarians	No replication of the association of *DUSP6*, *NALP1* and *PERP* with longevity
Nebel A, Kleindorp R, Caliebe A, Nothnagel M, Blanché H, Junge O, Wittig M, Ellinghaus D, Flachsbart F, Wichmann HE, Meitinger T, Nikolaus S, Franke A, Krawczak M, Lathrop M, Schreiber S	A genome-wide association study confirms APOE as the major gene influencing survival in long-lived individuals	Mech Ageing Dev	2011	132(6-7):324-30	Genetics	Research Article	536 centenarians	No association of ASTN1 and other markers with longevity, except *APOE*
Deelen J, Beekman M, Uh HW, Broer L, Ayers KL, Tan Q, Kamatani Y, Bennet AM, Tamm R, Trompet S, Guðbjartsson DF, Flachsbart F, Rose G, Viktorin A, Fischer K, Nygaard M, Cordell HJ, Crocco P, van den Akker EB, Böhringer S, Helmer Q, Nelson CP, Saunders GI, Alver M, Andersen-Ranberg K, Breen ME, van der Breggen R, Caliebe A, Capri M, Cevenini E, Collerton JC, Dato S, Davies K, Ford I, Gampe J, Garagnani P, de Geus EJ, Harrow J, van Heemst D, Heijmans BT, Heinsen FA, Hottenga JJ, Hofman A, Jeune B, Jonsson PV, Lathrop M, Lechner D, Martin-Ruiz C, Mcnerlan SE, Mihailov E, Montesanto A, Mooijaart SP, Murphy A, Nohr EA, Paternoster L, Postmus I, Rivadeneira F, Ross OA, Salvioli S, Sattar N, Schreiber S, Stefánsson H, Stott DJ, Tiemeier H, Uitterlinden AG, Westendorp RG, Willemsen G, Samani NJ, Galan P, Sørensen TI, Boomsma DI, Jukema JW, Rea IM, Passarino G, de Craen AJ, Christensen K, Nebel A, Stefánsson K, Metspalu A, Magnusson P, Blanché H, Christiansen L, Kirkwood TB, van Duijn CM, Franceschi C, Houwing-Duistermaat JJ, Slagboom PE	Genome-wide association meta-analysis of human longevity identifies a novel locus conferring survival beyond 90 years of age	Hum Mol Genet	2014	23(16):4420-32	Genetics	Meta-analysis	998 centenarians and 236 nonagenarians	Association of rs2149954 with longevity ($p = 1.74 \times 10^{-8}$) and survival beyond 90 years of age ($p = 0.003$) (meta-analysis)
Flachsbart F, Ellinghaus D, Gentschew L, Heinsen FA, Caliebe A, Christiansen L, Nygaard M, Christensen K, Blanché H, Deleuze JF, Derbois C, Galan P, Büning C, Brand S, Peters A, Strauch K, Müller-Nurasyid M, Hoffmann P, Nöthen MM, Lieb W, Franke A, Schreiber S, Nebel A	Immunochip analysis identifies association of the RAD50/IL13 region with human longevity	Aging Cell	2016	15(3):585-8	Genetics	Research Article/Meta-Analysis	1257 long-lived individuals (≥90 years old)	Replication of the association of SNP rs2706372 located in a region encompassing *RAD50* and *IL13* with longevity ($p = 2.69 \times 10^{-3}$)
Flachsbart F, Dose J, Gentschew L, Geismann C, Caliebe A, Knecht C, Nygaard M, Badarinarayan N, ElSharawy A, May S, Luzius A, Torres GG, Jentzsch M, Forster M, Häsler R, Pallauf K, Lieb W, Derbois C, Galan P, Drichel D, Arlt A, Till A, Krause-Kyora B, Rimbach G, Blanché H, Deleuze JF, Christiansen L,	Identification and characterization of two functional variants in the human longevity gene FOXO3	Nat Commun	2017	18(1):2063	Genetics	Research Article	1264 long-lived individuals (≥90 years old)	Replication of the associations for rs4946935 and rs12206094 in the *FOXO3* gene region with longevity ($p = 0.022$ and $p = 0.008$)

Christensen K, Nothnagel M, Rosenstiel P, Schreiber S, Franke A, Sebens S, Nebel A.								
Deelen J, Evans DS, Arking DE, Tesi N, Nygaard M, Liu X, Wojczynski MK, Biggs ML, van der Spek A, Atzmon G, Ware EB, Sarnowski C, Smith AV, Seppälä I, Cordell HJ, Dose J, Amin N, Arnold AM, Ayers KL, Barzilai N, Becker EJ, Beekman M, Blanché H, Christensen K, Christiansen L, Collerton JC, Cubaynes S, Cummings SR, Davies K, Debrabant B, Deleuze JF, Duncan R, Faul JD, Franceschi C, Galan P, Gudnason V, Harris TB, Huisman M, Hurme MA, Jagger C, Jansen I, Jylhä M, Kähönen M, Karasik D, Kardia SLR, Kingston A, Kirkwood TBL, Launer LJ, Lehtimäki T, Lieb W, Lyytikäinen LP, Martin-Ruiz C, Min J, Nebel A, Newman AB, Nie C, Nohr EA, Orwoll ES, Perls TT, Province MA, Psaty BM, Raitakari OT, Reinders MJT, Robine JM, Rotter JI, Sebastiani P, Smith J, Sørensen TIA, Taylor KD, Uitterlinden AG, van der Flier W, van der Lee SJ, van Duijn CM, van Heemst D, Vaupel JW, Weir D, Ye K, Zeng Y, Zheng W, Holstege H, Kiel DP, Lunetta KL, Slagboom PE, Murabito JM.	A meta-analysis of genome-wide association studies identifies multiple longevity genes	Nat Commun	2019	10(1):3669	Genetics	Research Article/Meta-Analysis	1234 long-lived individuals (≥90 years old)	Association of rs7676745 near GPR78 with longevity ($p = 4.3 \times 10^{-8}$) (meta-analysis)
Torres GG, Nygaard M, Caliebe A, Blanché H, Chantalat S, Galan P, Lieb W, Christiansen L, Deleuze JF, Christensen K, Strauch K, Müller-Nurasyid M, Peters A, Nöthen MM, Hoffmann P, Flachsbart F, Schreiber S, Ellinghaus D, Franke A, Dose J, Nebel A.	Exome-Wide Association Study Identifies FN3KRP and PGP as New Candidate Longevity Genes	J Gerontol A Biol Sci Med Sci	2021	76(5):786-795	Genetics	Research Article	1264 long-lived individuals (≥90 years old)	No replication of the SNVs associated with longevity, except for rs1063192 (*CDKN2B-AS1*) ($p = 0.0188$)
Daunay A, Hardy LM, Bouyacoub Y, Sahbatou M, Touvier M, Blanché H, Deleuze JF, How-Kit A.	Centenarians consistently present a younger epigenetic age than their chronological age with four epigenetic clocks based on a small number of CpG sites	Aging (Albany NY)	2022	14(19):7718-7733	Epigenetics	Research Article	214 centenarians and 143 offspring of centenarians and nonagenarians	Lower epigenetic age of centenarians and offspring of centenarians and nonagenarians compared to control individuals
